# Trypanosome infections in naturally infected horses and donkeys of three active sleeping sickness foci in the south of Chad

**DOI:** 10.1186/s13071-020-04192-1

**Published:** 2020-06-23

**Authors:** Joël Vourchakbé, Arnol Auvaker Z. Tiofack, Mpoame Mbida, Gustave Simo

**Affiliations:** 1 Molecular Parasitology and Entomology Unit, Department of Biochemistry, Faculty of Science, University of Dschang, PO Box 67, Dschang, Cameroon; 2 Department of Chemistry-Biology-Geology, Faculty of Science and Technology, University of Doba, PO Box 03, Doba, Chad; 3 Laboratory of Applied Biology and Ecology (LABEA), Department of Animal Biology, Faculty of Science, University of Dschang, PO Box 067, Dschang, Cameroon

**Keywords:** Human African trypanosomiasis, Donkeys, Horses, Trypanosomes, *Trypanosoma brucei gambiense*

## Abstract

**Background:**

Equine trypanosomiases are complex infectious diseases with overlapping clinical signs defined by their mode of transmission. Despite their economic impacts, these diseases have been neglected by the scientific community, the veterinary authorities and regulatory organizations. To fill the observed knowledge gap, we undertook the identification of different trypanosome species and subspecies naturally infecting horses and donkeys within the Chadian sleeping sickness focus. The objective of the study was to investigate the potential role of these domestic animals as reservoirs of the human-infective *Trypanosoma brucei gambiense.*

**Method:**

Blood samples were collected from 155 donkeys and 131 horses in three human African trypanosomiasis (HAT) foci in Chad. Rapid diagnostic test (RDT) and capillary tube centrifugation (CTC) test were used to search for trypanosome infections. DNA was extracted from each blood sample and different trypanosome species and subspecies were identified with molecular tools.

**Results:**

From 286 blood samples collected, 54 (18.9%) and 36 (12.6%) were positive for RDT and CTC, respectively. PCR revealed 101 (35.3%) animals with trypanosome infections. The Cohen’s kappa coefficient used to evaluate the concordance between the diagnostic methods were low; ranging from 0.09 ± 0.05 to 0.48 ± 0.07. Trypanosomes of the subgenus *Trypanozoon* were the most prevalent (29.4%), followed by *T. congolense* forest (11.5%), *Trypanosoma congolense* savannah (4.9%) and *Trypanosoma vivax* (4.5%). Two donkeys and one horse from the Maro HAT focus were found with *T. b. gambiense* infections. No significant differences were observed in the infection rates of different trypanosomes between animal species and HAT foci.

**Conclusions:**

This study revealed several trypanosome species and subspecies in donkeys and horses, highlighting the existence of AAT in HAT foci in Chad. The identification of *T. b. gambiense* in donkeys and horses suggests considering these animals as potential reservoir for HAT in Chad. The presence of both human-infective and human non-infective trypanosomes species highlights the need for developing joint control strategies for HAT and AAT.
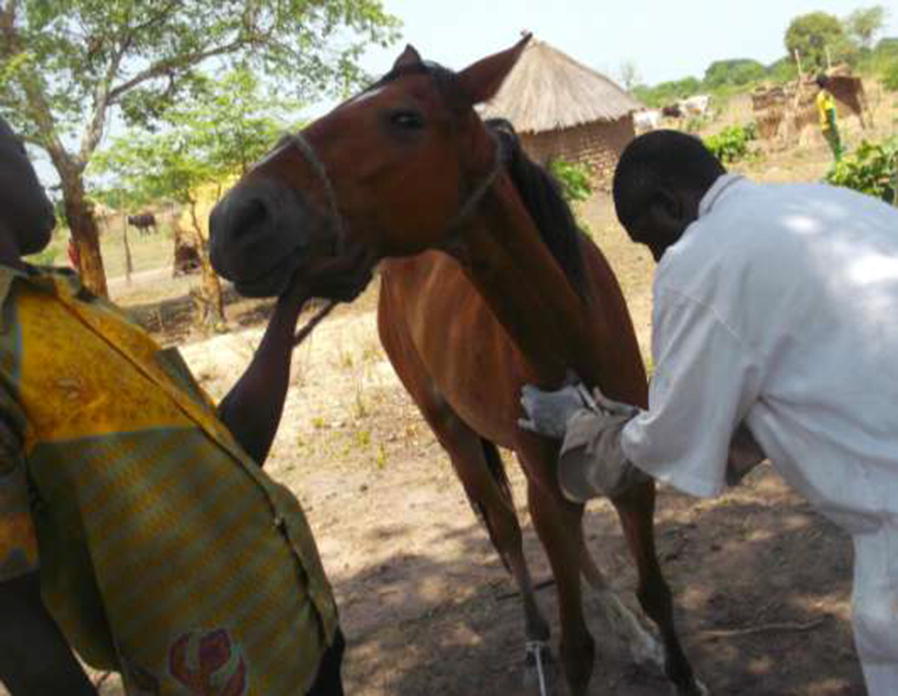

## Background

Trypanosomiases are infectious diseases affecting both humans and animals. Human African trypanosomiasis (HAT), also known as sleeping sickness, is an important public health disease caused by *Trypanosoma brucei gambiense* and *Trypanosoma brucei rhodesiense*. The former parasite causes the chronic form of HAT in west and central Africa while the latter induces an acute form which is found in eastern and southern Africa [1]. These human-infective parasites can be transmitted to livestock and wildlife, which can serve as reservoirs for HAT [2]. On the basis of the HAT-related mortality, HAT has been ranked ninth out of 25 human infectious and parasitic diseases in Africa [3, 4]. During the last three decades, efforts undertaken to fight HAT have brought this disease under control and led to its inclusion in the WHO “roadmap for eradication, elimination and control of neglected tropical diseases”, with a target set to eliminate HAT as a public health problem by 2020 [5]. Achieving these goals requires investigation of animal reservoirs, which have been considered as one component that could compromise the elimination and eradication of HAT. Several trypanosome species and subspecies including *T. b. gambiense* were reported in various animal species of western and central African HAT foci. Although some investigations have been undertaken on trypanosome infections in donkeys and horses of AAT endemic areas of West Africa [6–8], such data are lacking in HAT foci of central Africa despite the fact these animals are commonly used by inhabitants for traction and transport. However, these animals are exposed to trypanosome infections and could alter the dynamics of HAT infection, thus jeopardize eradication efforts.

African animal trypanosomiases (AAT) are responsible of major constraints to livestock production in affected countries. Their direct impact is linked to the reduction of livestock productivity, while the indirect impacts are associated with a reduced efficiency of draught animals for crop production [9, 10]. Although several trypanosome species have been reported in domestic and wild animals residing within HAT foci in west and central Africa [11–16], equines (mules, donkeys and horses) have not been addressed thus far. Indeed, the equine population is estimated to be more than 127 million with approximately 85% in low income countries [17]. The positive impact of equines has been widely acknowledged upon poverty reduction, gender equality and environmental stability [18, 19]. Equines maintain the health and welfare of 300 to 600 million people globally, often within the most vulnerable communities [20]. They play an important role in transport and traction [21], contribute significantly to household income [22] and create opportunities for women and children [23]. Due to their importance, attempts have been refocused to tackle infectious diseases that could compromise the welfare and productivity of these animals [17–20]. In this light, equine trypanosomiasis was reported as one of the infectious diseases that may have the greatest impact upon working equines [10].

Equine trypanosomiasis caused by species of the genus *Trypanosoma* is a complex of infectious diseases called dourine, nagana and surra. These diseases are characterized by overlapping clinical features that can be defined by their mode of transmission [23]. They give rise to important economic losses in Africa, the Middle East, Asia and Latin America [24]. They can be considered as animal diseases that are seriously neglected, both by the scientific community and by veterinary authorities and regulatory organizations [24]. Nagana is caused by *T. vivax*, *T. congolense* and/or *T. brucei* subspecies and is transmitted by tsetse flies; surra is caused by *T. evansi* and is mechanically transmitted by biting flies; while dourine is due to *T. equiperdum* and is sexually transmitted [23]. With these transmission modes, designing appropriate control measures requires a better understanding of the epidemiology of equine trypanosomiasis by identifying trypanosomes that naturally infect horses and donkeys. In HAT foci, such investigation may generate data for the improvement of epidemiological knowledge on AAT and animal reservoirs of HAT.

The present study was designed to identify trypanosome species in naturally infected horses and donkeys of three active sleeping sickness foci in Chad and to assess if these animals can serve as reservoir hosts for *T. b. gambiense*.

## Methods

### Study sites

**Fig. 1:**
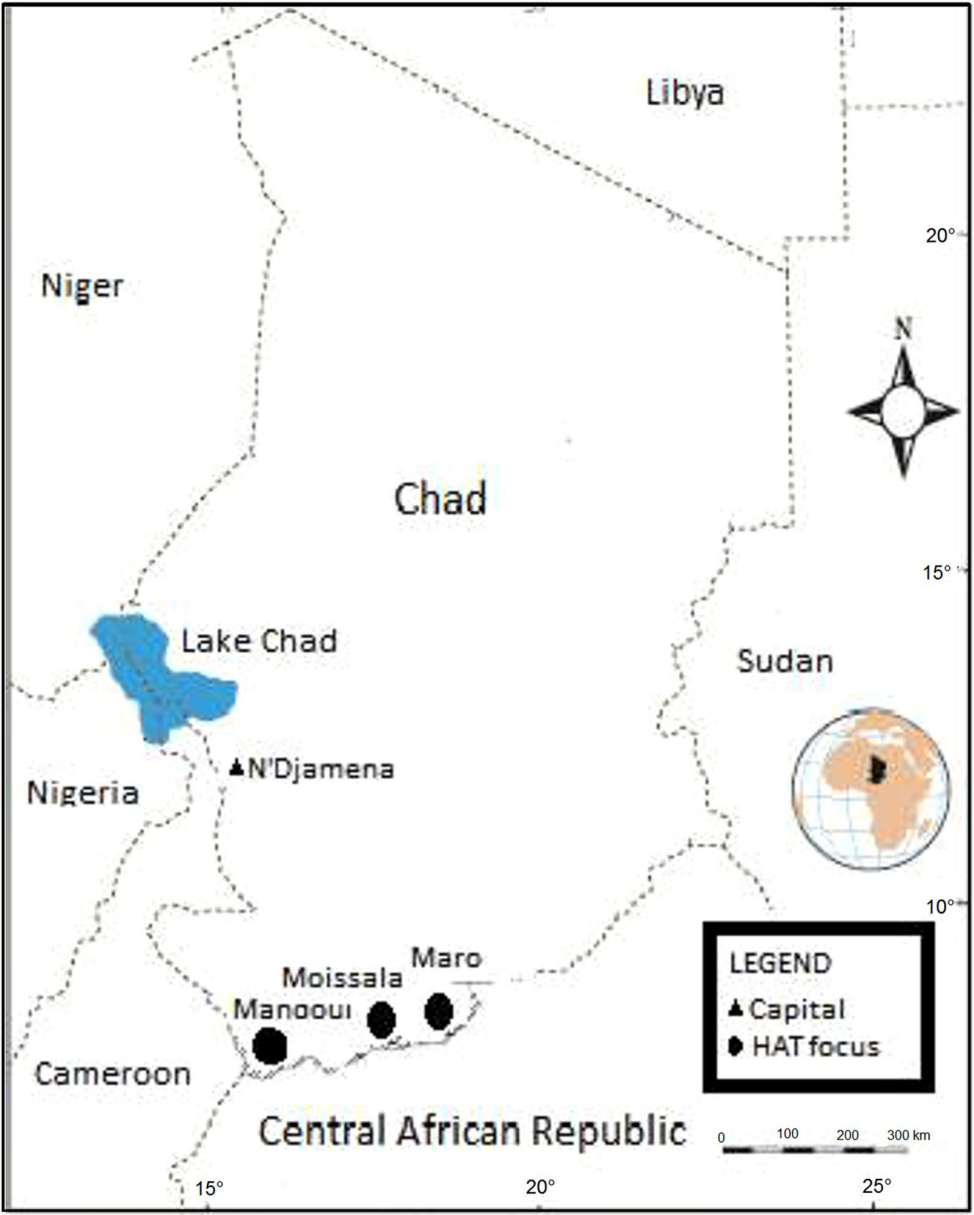
Map showing sleeping sickness foci where donkeys and horses were sampled in the south of Chad

This cross-sectional study was conducted in three active HAT foci located in the extreme southern part of Chad. These HAT foci include the Maro, Mandoul and Moissala (Fig. 1).

The HAT focus of Mandoul (8°6′57″N, 17°06′58″E) was previously called the Bodo HAT focus [25]. Located at the borders of Cameroon and the Central African Republic, the HAT focus of Mandoul is *c.*50 km from Doba, the capital of the “Logone Oriental” region. It has 45 villages and belongs to areas showing a low risk for HAT [26, 27]. Its temperature varies between 22 and 38 °C and the average annual rainfall is 1000 mm [28]. The landscape is mainly dominated by forest galleries and wooded savannah that provide favorable conditions for tsetse flies such as *Glossina tachinoides*, *G. fuscipes fuscipes* and *G. morsitans submorsitans*, previously reported in the south of Chad [26]. The inhabitants of this focus practice peasant farming around the forest galleries where they build their huts or houses. The main agricultural activities are cotton, millet and sesame cultivation. Inhabitants also practice extensive animal breeding (cattle, sheep, goats, pigs and some equines). During the dry season, the Mandoul River offers a meadow to many Bororos herders in transhumance.

The Mara HAT focus (8°28′33″N, 18°46′10″E) is located at 55 km from Sarh, the capital of the “Moyen Chari” region. It is located at the border of the Central African Republic and contains 33 villages. It belongs to foci showing a moderate risk for HAT [27]. Most of these villages are located near the great Sido River. Its temperature varies between 25–38 °C and precipitation varies between 800–1300 mm. The vegetation is made up of savannah and clear forests with dotted trees. This vegetation offers favorable environmental conditions for the reproduction and survival of tsetse flies. Inhabitants of this HAT focus practice peasant agriculture with millet and cassava cultivation being the most predominant agricultural activities. They also practice fishing, gathering, hunting and animal breeding (cattle, sheep, goats, pigs and horses). The presence of nomadic pastoralists such as Bororo and Arabs leads to a very large cross-border movement of populations between Chad and the Central African Republic.

The Moissala HAT focus (8°20′25″N, 17°45′58″E) is part of the great historical HAT focus of Middle Chari [25]. It extends on the left and the right sides of Nana-Barya River and between Bahr Sara (Ouham) and Chari rivers. It is located in the South of Koumra, the capital of the Mandoul region, within *c.*400 km of the Central Africa Republic border. It has 25 villages and belongs to foci showing a moderate risk for HAT [27]. The temperature varies between 24–38 °C and the average annual rainfall is about 1100 mm. The vegetation is formed by forest galleries which offer favorable conditions for the reproduction and development of tsetse flies. Inhabitants of this HAT focus practice peasant agriculture dominated by cotton, millet and sesame cultivation. They also practice extensive animal breeding (cattle, sheep, goats, pigs and some equines).

In the three HAT foci, the majority of inhabitants were traditional small farm holders practicing small scale animal husbandry. Sheep and goats are usually reared together with cattle. Donkeys and horses are commonly used for transportation and traction. The grazing system is essentially free grazing.

### Sample collection, immunological and parasitological analyses

Donkeys and horses were sampled during field surveys in three active HAT foci in Chad. Two surveys (2018 and 2019) were carried out from April to May. Before each survey, the objective of the study was re-explained to inhabitants and local authorities of the villages. One day before the sampling, the inhabitants were asked to restrain and/or keep their animals. In each village, all donkeys and horses that had spent at least 3 months in the study zone were selected. From each animal, *c.*5 ml of blood was collected into EDTA coated tubes; collection was performed from the jugular vein in horses and donkeys. The tubes were labelled and carefully packed to avoid cross contamination. All horses sampled in this study were of the “Poney du Logone” or “Poney Musey” breed, while donkeys were a local breed [29].

Capillary tube centrifugation test, as described by Woo [30], was performed on each blood sample to search for trypanosomes. To identify animals that had been in contact with *T. b. gambiense*, the gHAT rapid diagnostic test (RDT) was performed in parallel as described by Matovu et al. [31]. The RDT named SD BIOLINE HAT was used in this study. It was developed using native VSGs (Nat-LiTat 1.3 and Nat-LiTat 1.5) obtained from the Institute of Tropical Medicine (ITM) in Antwerp, Belgium [31]. It detects anti-VSG LiTat 1.3 and anti-VSG LiTat 1.5 antibodies [32–34].

On completion of the immunological and parasitological tests, remaining blood samples were centrifuged at 13,000× *rpm* for 5 min. The buffy coat was transferred into 1.5 ml micro-tubes, stored in an electric cooler and transported to the Molecular Parasitology and Entomology Unit of the Department of Biochemistry of the Faculty of Science of the University of Dschang, Cameroon. They were stored at − 20 °C until DNA extraction for molecular analyses.

During sample collection, each animal was examined by a veterinarian and its clinical status was recorded.

### Extraction of genomic DNA

Genomic DNA was extracted from each buffy coat sample using the cethyl trimethyl ammonium bromide (CTAB) method. Briefly, 500 µl of buffy coat and 1 ml of nuclease-free water were mixed in a 2 ml micro-tube. The mixture was vigorously homogenized and then centrifuged at 11,000× *rpm* for 15 min. The supernatant was removed and 600 µl of CTAB buffer (CTAB at 5%; 1 M Tris, pH 8.0; 0.5 M EDTA, pH 8.0; 5 M NaCl) was added to the resulting pellet. The latter was re-suspended and incubated in a water bath at 60 °C for 30 min. Once cooled, 600 µl of chloroform/isoamyl alcohol (24/1) mixture was added to the contents of each micro-tube. Each micro-tube was slowly homogenized for 15 min and the upper aqueous phase was removed and transferred to a new 1.5 ml micro-tube. DNA was precipitated by adding 600 µl of isopropanol. The mixture was gently homogenized for 5 min and then incubated overnight at − 20 °C. After this incubation, each micro-tube was centrifuged at 13,000× *rpm* for 15 min. The DNA pellet was then washed twice with cold 70% ethanol and dried overnight at room temperature. The resulting DNA pellet was re-suspended in 50 µl of sterile nuclease-free water and stored at − 20 °C until use.

### Molecular identification of different trypanosome species

Trypanosome identification was achieved by amplifying the internal transcribed spacer 1 (ITS1) of ribosomal DNA of different trypanosome species as described by Ravel et al. [35]. For this identification, two PCR rounds were performed; the first round was carried out in a final volume of 25 µl containing 1× PCR buffer (10 mM Tris-HCl (pH 9.0), 50 mM KCl), 2 mM MgCl_2_, 1 µl (10 pmol) of each primer (5′-CAA ATT GCC CAA TGT CG-3′ and 5′-GCT GCG TTC TTC AAC GAA-3′), 0.5 µl (200 mM) of dNTPs, 1 µl (one unit) of Taq DNA polymerase (5 U/µl; New England Biolabs, Ipswich, Massachusetts, USA), 5 μl of DNA and 14 µl of nuclease free water. The amplification program began with a denaturation step at 94 °C for 3 min and 30 s followed by 30 amplification cycles; each of these cycles contained a denaturation step at 94 °C for 30 s, an annealing step at 58 °C for 1 min, and an extension step at 72 °C for 1 min, followed by a final extension step at 72 °C for 5 min.

The amplified products of the first PCR round were diluted 10-fold and 3 µl of each dilution was used as template for the second PCR round. The second PCR round was performed with two different primers (5′-CCT GCA GCT GGA TCA T-3′ and 5′-ATC GCG ACA CGT TGT G-3′). The amplification program was identical to that of the first PCR round. After the nested PCR, amplicons were separated by electrophoresis on a 2% agarose gel that was subsequently stained with ethidium bromide and visualized under UV light.

Different trypanosome species were identified based on the length polymorphism of their ITS1 fragments. For instance, *T. congolense* strains generate DNA fragments of around 650 bp (630 bp for *T. congolense* forest and 610 bp for *T. congolense* savannah) while fragments of about 150 bp and 400 bp, respectively are expected for *T. vivax* and all trypanosomes belonging to the subgenus *Trypanozoon* (*T. brucei* (*s.l*.), *T. evansi* and *T equiperdum*).

### Identification of *Trypanosoma congolence* forest and *Trypanosoma congolence* savannah

Following the amplification of ITS1 sequences, all samples that had a DNA fragment between 600–650 bp, corresponding to the expected size of *T. congolense*, were subjected to another PCR where specific primers were used to identify *T. congolence* forest “type” or *T. congolence* savannah “type”. These specific identifications were performed as described by Simo et al. [11] using the primers TCF_1_ (5′-GGA CAC ACG CCA GAA GGT ACT T-3′) and TCF_2_ (5′-GTT CTC TCG CAC CAA ATC CAA C-3′) for *T. congolence* forest “type” [36], and TCS_1_ (5′-CGA GCG AGA ACG GGC AC-3′) and TCS_2_ (5′-GGG ACA AAC AAA TCC CGC-3′) for *T. congolense* savannah “type” [37]. PCR reactions were carried out in a final volume of 25 μl containing 1× PCR buffer (10 mM Tris-HCl (pH 9.0), 50 mM KCl), 3 mM MgCl_2_, 1 µl (15 pmol) of each primer, 0.5 μl (200 mM) of dNTPs, 1 μl (one unit) of Taq DNA polymerase, 3 µl of DNA and 16 μl of sterile water. The amplification program comprised a denaturation step at 94 °C for 3 min 30 s, followed by 40 amplification cycles that included a denaturation step at 94 °C for 30 s, a hybridization step at 60 °C for 1 min and elongation step at 72 °C for 1 min, followed by a final elongation step at 72 °C for 5 min.

The amplified products were separated by electrophoresis on a 2% agarose gel containing ethidium bromide (0.3 μg/ml). The DNA bands were visualized under ultraviolet (UV) light and then photographed.

### Identification of *Trypanosoma brucei gambiense*

Identification of *T. b. gambiense* was only performed on samples that had a DNA fragment of *c.*400 bp, corresponding to the expected size of trypanosomes belonging to the subgenus *Trypanozoon* (*T. b. brucei*, *T. evansi*, *T. b. gambiense* and *T. b. rhodesiense*). On these samples, *T. b. gambiense* was identified as described by Cordon-Obras et al. [15]. This was achieved using a nested PCR with two pairs of primers specific to *T. b. gambiense.* The primer pairs TgSGP1 (5′-GCT GCT GTG TTC GGA GAG C-3′ and TgSGP2- (5′-GCC ATC GTG CTT GCC GCT C-3′) described by Radwanska et al. [38], and TgsGPs (5′-TCA GAC AGG GCT GTA ATA GCA AGC-3′) and TgsGPas (5′-GGG CTC CTG CCT CAA TTG CTG CA-3′) designed by Morrison et al. [39] were used.

The first PCR round was carried out in a total volume of 25 µl containing 2.5 µl of 10× PCR buffer (10 mM Tris-HCl (pH 9.0), 50 mM KCl, 3 mM MgCl_2_), 1 µl (15 pmol) of each of primer (TgSGP1 and TgSGP2), 0.5 µl (100 mM) of dNTPs, 1 µl (one unit) of Taq DNA polymerase, 5 μl of DNA and 14 μl of sterile water. The amplification program comprised an initial denaturation step at 95 °C for 3 min, followed by 45 cycles of 95 °C for 30 s, 63 °C for 1 min and 72 °C for 1 min, and a final elongation step at 72 °C for 5 min. Amplified products of the first PCR round were diluted 1:10 in sterile water and 5 µl of each dilution was used as the DNA template for the second round PCR. The primers TgsGPs and TgsGPas were used and only 25 amplification cycles were performed using the same conditions as for the first PCR round.

The amplified products were separated by electrophoresis on a 2% agarose gel containing ethidium bromide (0.3 μg/ml). DNA bands were visualized under UV light and then photographed.

### Data analyses

Statistical analyses were performed to compare the trypanosome infection rates between animal species and HAT foci using XLSTAT 2016 software (https://www.xlstat.com/en/). The Chi-square test was used to compare the infection rates of different trypanosomes between animal species and different HAT foci. The threshold for significance was set at below 5%. To estimate the concordance between results generated by the tests used to identify different trypanosome infections, the kappa coefficient was determined according to Cohen [40], and interpreted as described by Altman [41].

## Results

### Results of parasitological (CTC) and immunological (RDT) tests

For this study, 286 animals including 155 (54.2%) donkeys and 131 (45.8%) horses were sampled in the three HAT foci in Chad. Forty-seven (16.4%) animals including 30 (10.5%) donkeys and 17 (5.9%) horses were from the Mandoul HAT focus, 180 (62.9%) including 84 (29.4%) donkeys and 96 (33.6%) horses were from the Maro HAT focus and 59 (20.6%) animals including 41 (14.3%) donkeys and 18 (6.3%) horses were from the Moissala HAT focus (Table 1). Comparing results of RDT between the three HAT foci, slight variations were observed without any significant difference (*χ*^2^ = 0.19, *df* = 2, *P* = 0.91) (Table 1). Similarly, no statistically significant difference was observed in the trypanosome infection rates despite a little variation between HAT foci for the parasitological tests (CTC) (*χ*^2^ = 0.04, *df* = 2, *P* = 0.98) (Table 1).

**Table 1:** Trypanosome infections according to HAT foci

HAT foci	NE		RDT+	T+	PCR results						
	Donkey	Horse			TB	TC+	TCS+	TCF+	TV+	TBG+	Total^a^
Mandoul	30	17	8 (17.0%)	6 (12.8%)	17 (36.2%)	8 (17.0%)	1 (2.1%)	7 (14.9%)	3 (6.4%)	0	19 (40.4%)
Maro	84	96	34 (18.9%)	23 (12.8%)	49 (27.2%)	31 (17.2%)	9 (5.0%)	22 (12.2%)	5 (2.8%)	3 (1.7%)	60 (33.3%)
Moissala	41	18	12 (20.3%)	7 (11.9%)	18 (30.5%)	8 (13.6%)	4 (6.8%)	4 (6.8%)	5 (8.5%)	0	22 (37.3%)
Total	155	131	54 (18.9%)	36 (12.6%)	84 (29.4%)	47 (16.4%)	14 (4.9%)	33 (11.5%)	13 (4.5%)	3 (1.04%)	101 (35.3%)
*χ*^2^			0.19	0.04	1.48	0.45	1.28	1.91	3.76	1.79	
*P*-value			0.91	0.98	0.48	0.80	0.541	0.385	0.15	0.41	

^a^The numbers contained in this column are lower than the sum of TB^+^, TC^+^ and TV^+^ because some animals were carriers of mixed infections

*Abbreviations*: NE, number of animals examined; RDT, rapid diagnosis test; T+: trypanosome infections revealed by capillary tube centrifugation; TB, trypanosomes belonging to the subgenus *Trypanozoon* (includes TBG); TC, *Trypanosoma congolense* (includes TCF and TCS); TCS, *Trypanosoma congolense* savannah type; TCF, *Trypanosoma congolense* forest type; TV, *Trypanosoma vivax*; TBG, *Trypanosoma brucei gambiense*

Of the 286 equines, 54 (18.9%) were positive by RDT: 32 (20.6%) donkeys and 22 (16.8%) horses (Table 2). The number of horses and donkeys positive by RDT did not differ significantly (*χ*^2^ = 0.69, *df* = 1, *P* = 0.41) (Table 1). The parasitological test (CTC) revealed trypanosomes in 36 (12.6%) animals: 22 (14.2%) donkeys and 14 (10.7%) horses. No significant difference (*χ*^2^ = 0.79, *df* = 1, *P* = 0.37) between donkeys and horses was observed in relation to trypanosome infection rates (Table 2).

**Table 2:** Trypanosome infections according to animal species

Animal species	NE	RDT+	T+	PCR results						
				TB	TC+	TCS+	TCF+	TV+	TBG+	Total^a^
Donkeys	155	32 (20.6%)	22 (14.2%)	49 (31.6%)	27 (17.4%)	8 (5.2%)	19 (12.3%)	6 (3.9%)	2 (1.3%)	61 (39.3%)
Horses	131	22 (16.8%)	14 (10.7%)	35 (26.7%)	20 (15.3%)	6 (4.6%)	14 (10.7%)	7 (5.3%)	1 (0.8%)	40 (30.5%)
Total	286	54 (18.9%)	36 (12.6%)	84 (29.4%)	47 (16.4%)	14 (4.9%)	33 (11.5%)	13 (4.5%)	3 (1.0%)	101 (35.3%)
*χ*^2^	–	0.69	0.79	0.82	0.24	0.05	0.17	0.35	0.19	
*P*-value	–	0.41	0.37	0.37	0.62	0.82	0.68	0.55	0.66	

^a^The numbers contained in this column are lower than the sum of TB^+^, TC^+^ and TV^+^ because some animals were carriers of mixed infections

*Abbreviations*: NE, number of animals examined; RDT, rapid diagnosis test; T+: trypanosome infections revealed by capillary tube centrifugation; TB, trypanosomes belonging to the subgenus *Trypanozoon* (includes TBG); TC, *Trypanosoma congolense* (includes TCF and TCS); TCS, *Trypanosoma congolense* savannah type; TCF, *Trypanosoma congolense* forest type; TV, *Trypanosoma vivax*; TBG, *Trypanosoma brucei gambiense*

### Molecular detection of different trypanosomes

**Fig. 2:**
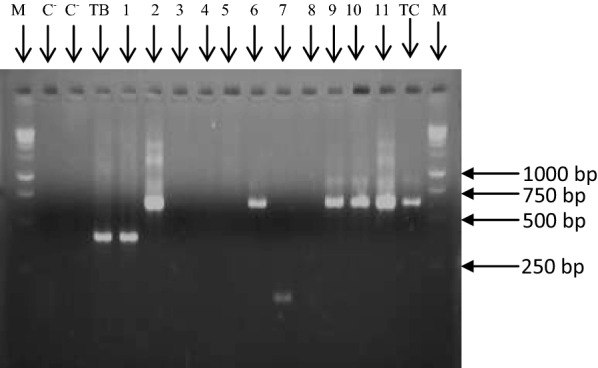
Electrophoretic profiles illustrating DNA fragments resulting from the amplification of ITS1 of different trypanosome species. Lanes C−: negative controls; Lane TB+: positive control of purified DNA of *T. b. gambiense* isolate; Lane TC: positive control of purified DNA of *T. congolense* forest isolate; Lane M: molecular marker (GeneRuler1 kb DNA ladder); Lane 1: sample with trypanosomes of the subgenus *Trypanozoon*; Lanes 2, 6, 9, 10 and 11: samples *T. congolense* infections; Lane 7: sample with *T. vivax*; Lanes 3, 4, 5 and 8: samples with no trypanosome infection

In this study, a variety of trypanosome species and subspecies including *T. vivax*, *T. congolense* forest and savannah, and trypanosomes belonging to the subgenus *Trypanozoon* were identified (Fig. 2). Trypanosome DNA was found in 101 out of 286 animals examined (Tables 1, 2) resulting in an overall prevalence of 35.3% (101/286): 39.3% (61/155) in donkeys and 30.5% (40/131) in horses (Table 2). At the species level, *T. vivax* had the lowest infection rate (4.5%) followed by *T. congolense* (16.4%). Amongst the 47 animals found with *T. congolense* infections, 33 (70.2%, 33/47) were due to *T. congolense* forest and 14 (29.8%, 14/47) to *T. congolense* savannah. This gives an overall infection rate of 11.5% for *T. congolense* forest and 4.9% for *T. congolense* savannah. No significant difference was found in the trypanosome infection rates between horses and donkeys, neither for *T. vivax* nor for different *T. congolense* subspecies (Table 2).

Trypanosomes belonging to the subgenus *Trypanozoon* (*T. evansi*, *T. equiperdum* and *T. brucei*) were found with the highest infection rate of 29.4% (84/286). No significant difference (*χ*^2^ = 0.82, *df* = 1, *P* = 0.37) was found in the infection rates of trypanosomes of the subgenus *Trypanozoon* between horses and donkeys (Table 2). The primers used to identify trypanosomes were not able to differentiate trypanosomes of the subgenus *Trypanozoon*. Therefore, the 84 animals identified with a DNA fragment with the molecular size of trypanosomes of the subgenus *Trypanozoon*, could be infected with *T. brucei* (*s.l*.), *T. evansi*, *T. equiperdum* or a mixture of two or three of these subspecies.

Between HAT foci, the trypanosome infection rates varied slightly without any significant difference (Table 1). The highest trypanosome infection rate of 40.4% (19/47) was observed in the Mandoul HAT focus (Table 1). No significant difference was found in the infection rates of different trypanosome species and subspecies despite some slight variations observed between HAT foci (Table 1).

Out of 101 animals infected by trypanosomes, mixed infections of different trypanosome species were found in 36 (35.6%, 36/101) of them: 19 (31.2%, 19/61) donkeys and 17 (42.5%, 17/40) horses. Amongst the 286 animals analyzed in this study, 12.6% (36/286) harbored mixed infections of different trypanosome species: 12.3% (19/155) of donkeys and 13% (17/131) of horses. Of the 36 animals found with mixed infections, 29 harbored double infections and 7 triple infections. Twenty-six (89.7%, 26/29) double infections (14 in donkeys and 12 in horses) were a mixture of *T. congolense* with trypanosomes of the subgenus *Trypanozoon*. The remaining double infections (10.3%, 3/29) were found in donkeys and contained one mixed infection of *T. vivax* with trypanosomes of the subgenus *Trypanozoon* and two *T. congolense* + *T. vivax*.

Triple infections were found in 1.5% (2/131) of donkeys and 3.2% (5/155) of horses. They included *T. congolense*, *T. vivax* and trypanosomes of the subgenus *Trypanozoon*. Such mixed infections were underestimated given that mixed infections could occur amongst trypanosomes of the subgenus *Trypanozoon*.

### Molecular identification of *T. b. gambiense*

**Fig. 3:**
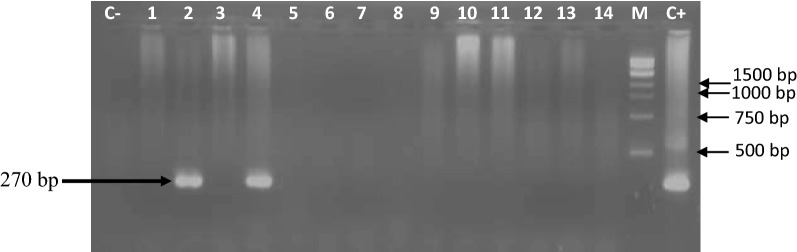
Electrophoretic profiles showing specific DNA fragments of *T. b. gambiense* that were amplified from donkeys and horses. Lane C−: negative control; Lane C+: positive control of purified DNA of *T. b. gambiense* isolate; Lane M: molecular marker (1 kb ladder); Lanes 2 and 4: samples with *T. b. gambiense* infections; Lanes 1, 3, 5–14: samples without *T. b. gambiense* infection, but harboring other trypanosomes of the subgenus *Trypanozoon*

The two sets of primers used to identify *T. b. gambiense* infections enabled amplification of a DNA fragment of 270 bp, which is specific to this *T. brucei* subspecies (Fig. 3). Of the 84 samples found with trypanosomes of the subgenus *Trypanozoon*, three animals were identified with infections due to *T. b. gambiense*; 2 donkeys and one horse (Table 2). This gives an overall infection rate of 1.0% (3/286) for *T. b. gambiense*: 1.3% (2/155) in donkeys and 0.8% (1/131) in horses. No significant difference (*χ*^2^ = 0.19, *df* = 1, *P* = 0.66) was found between *T. b. gambiense* infections in donkeys and horses. Remarkably, *T. b. gambiense* infections were found only in three animals of the Maro HAT focus resulting in an overall infection rate of 1.7% (3/180). This *T. brucei* subspecies was not found in animals sampled in the Mandoul and Moissala HAT foci (Table 1).

### Concordance between RDT, CTC and PCR for the detection of *T. b. gambiense*

From the 286 animals examined in this study, 54 were positive by using RDT, 36 by CTC and 101 by PCR. Concordant results between RDT and CTC were reported for 246 (86.0%, 246/286) samples: 25 (10.2%, 25/246) and 221 (89.8%, 221/246) samples, respectively, were positive and negative for both tests. Eleven samples were RDT−/CTC+ while 29 were RDT+/CTC− (Additional file 1: Table S1). Between RDT and CTC, the value of the concordance index, expressed here as the Cohen’s kappa coefficient, was 0.48 ± 0.0698 (95% CI: 0.339–0.6132), indicating a moderate strength of agreement.

Regarding the RDT and PCR targeting all trypanosome species, these tests were concordant for 207 (71.6%) samples: 38 (13.3%) and 169 (59.1%) samples, respectively, were positive and negative for both tests (Additional file 2: Table S2). The value of the Cohen’s kappa coefficient was 0.211 ± 0.045 (95% CI: 0.061–0.239); indicating a poor strength of agreement between RDT and PCR.

Between RDT and PCR detecting *T. b. gambiense*, these tests were concordant for 235 (82.2%) samples: 3 (1.04%) and 232 (81.1%) samples, respectively were positive and negative for both tests (Additional file 3: Table S3). The value of Cohen’s kappa coefficient was 0.087 ± 0.047 (95% CI: − 0.0056 to 0.1798). These results also indicate a poor strength of agreement between these tests.

## Discussion

To address the problem linked to African trypanosomiases, considerable efforts have been undertaken to identify trypanosomes in tsetse and various animals of HAT foci in west and central Africa. Despite data generated on trypanosome infections in animals from these foci, equine trypanosomiasis has not been addressed. It is to fill this knowledge gap that the presence of different trypanosome species was investigated in horses and donkeys from three HAT foci in Chad. This first study on equine trypanosomiasis in central African HAT foci revealed several trypanosome species and subspecies including *T. congolense*, *T. vivax* and trypanosomes of the subgenus *Trypanozoon* in donkeys and horses. Our results are in agreement with those reporting such infections in donkeys and horses of AAT-endemic areas of West Africa [6–8, 42].

The high infection rate of 35.3% revealed by PCR-based methods compared to 12.6% obtained with the CTC test suggests that most animals were infected by trypanosomes with low parasitaemia that was below the detection threshold of the CTC. Although the present overall infection rate of 35.3% is lower than 91% reported by Pinchbeck et al. [7] in West Africa, this value is within the range of previously published data which varied from 7% [8] to 40% [6]. The differences reported by these studies could be explained by the methods used to identify trypanosomes, the sampling sites and the population of investigated animals. Previously published data are largely based on microscopy which, from this study and previous research [6, 8], exhibits a much lower sensitivity compared to 1–20 trypanosomes/ml for PCR-based methodology [43, 44]. In studies reporting prevalence of above 90%, the majority of animals were clinically unhealthy and consequently more likely to be infected with trypanosomes [7, 8].

The RDT used here is an immune-chromatographic test for the screening of HAT and is expected to be positive only if the host has been in contact with *T. b. gambiense* [45]. The seroprevalence of 18.9% revealed here is too high, since only 3 (1.04%) animals were found with *T. b. gambiense* infections. The low specificity of RDT is in line with observations by Matovu et al. [31] reporting similar specificity in animals from an AAT-endemic region. It may result from the fact that the antigens used in RDTs could cross-react with epitopes of other trypanosome species, but are probably not predominant in these species [31]. This is not surprising if we consider the high similarity reported at the genomic level between different trypanosome species [46, 47]. As already reported in cattle [31], RDT used for specific identification of *T. b. gambiense* does not seem to be appropriate for horses and donkeys. This hypothesis is strengthened by the low Cohen’s kappa coefficient indicating the low strength of agreement between RDT and PCR used to identify *T. b. gambiense* in animals. The Cohen’s kappa coefficient remained low between the RDT and CTC tests, as well as RDT and PCR, also indicating a low or moderate agreement between these tests. The discrepancies between these tests could be partially explained by the fact that a positive PCR can be inferred as an active infection, while a positive RDT could be a current or past infection. All these results point to the fact that the antigens used in RDT may cross-react with other antigens not yet identified.

The infection rate (35.3%) reported here is higher than 27.1% and 18.7%, respectively, reported in domestic and wild animals of other Central African HAT foci [9, 11]. These differences may result from the animal species and the transmission patterns in each setting. In the present study, horses and donkeys were investigated, while pigs, sheep, goats, primates, rodents, carnivores and pangolins were analyzed in other studies [9, 11]. Moreover, the environmental conditions in the HAT foci in Chad are different from those of the HAT foci in the forest regions. In such contexts, the trypanosomes’ transmission will vary in response to the diversity of tsetse fauna.

The identification of *T. congolense* forest and savannah, *T. vivax* and trypanosomes of the subgenus *Trypanozoon* indicates the presence of AAT in the HAT foci in Chad. The higher infection rate reported in donkeys (39.3%) than horses (30.5%) contradicts results obtained elsewhere [8, 48]. In general, horses are considered more susceptible to trypanosome infections than donkeys [8]. Although the reasons explaining the high susceptibility of donkeys are still unknown, we can speculate on: (i) the nutritional behavior of vector populations; (ii) the density of biting flies that are responsible for the mechanical and cyclical transmission of trypanosomes; and (iii) the behavior of animal species in each epidemiological setting. These factors could interfere with trypanosomes’ transmission and consequently, infection rate. The absence of a significant difference in the prevalence of any trypanosome species suggests a similar transmission pattern of trypanosomes in horses and donkeys, and also in different HAT foci. This could be explained by the fact that horses and donkeys are used for the same purposes and hence, are exposed to similar levels of trypanosome transmission. Entomological investigations on tsetse (blood meal analysis and dynamics of tsetse populations) and other biting arthropods could enable determination of their nutritional behavior in relationship to horses and donkeys and consequently, the probability for each animal to acquire trypanosome infections.

Although molecular tests enabled, with relatively high sensitivity and excellent specificity, to identify *T. congolense*, *T. vivax* and *Trypanozoon* taxa, no single test is able to differentiate unequivocally trypanosomes of the subgenus *Trypanozoon* [24]. The presence of tsetse flies in the HAT foci in Chad indicates that some trypanosomes belonging to the subgenus *Trypanozoon* may be due to *T. brucei* (*s.l.*). This hypothesis is strengthened by the identification of *T. b. gambiense* in animals of one HAT focus. The geographical localization of these HAT foci does not exclude the possibility of having, in addition to *T. vivax*, *T. evansi* infections that can be mechanical transmitted by some biting flies [49]. This hypothesis is more plausible with previous identification of mechanical vectors such as Stomoxyinae and Tabanidae in these HAT foci [26]. Investigations on the nutritional behavior of biting flies could enable a better understanding of trypanosome transmission in each HAT focus. The slightly higher prevalence of trypanosomes of the subgenus *Trypanozoon* in donkeys (31.6%) compared to horses (26.7%) is in agreement with results obtained in the Gambia [8]. In addition, the slightly lower prevalence of *T. vivax* infections in donkeys (3.9%) than in horses (5.3%) corroborates also results of Pinchbeck et al. [8]. Understanding these differences requires investigation of the nutritional behavior of different biting flies.

Compared with *T. congolense* prevalence of 64% reported by Dhollander et al. [6], our low prevalence of 16.4% could be explained by the fact that the majority of animals previously investigated were anaemic, and consequently were more likely to carry trypanosome infections. The co-existence of *T. congolense* forest and savannah indicates that the geographical limit (*T. congolense* savannah and forest in the savannah and forest zones, respectively) tends to change with time. The high infection rate of *T. congolense* forest (12.9%) compared to *T. congolense* savannah (4.9%) could be explained by the geographical localization of most HAT foci in the forest galleries.

In addition to single-species infections, the present study showed that approximately 12.6% (36/286) of animals (12.3% of donkeys and 13.0% of horses) carried mixed infections comprised of different trypanosome species and subspecies. These results are in agreement with previous observations highlighting that mixed infections may be more frequent where several species co-exist [6, 8]. It is important to point out that these mixed infections are probably underestimated because some mixed infections could exist between trypanosomes (*T. evansi*, *T. equiperdum* and *T. brucei* (*s.l*.)) of the subgenus *Trypanozoon*. With the high number of double and triple infections reported in this study, there is a need to understand their evolution and their potential impacts on animal health, and the transmission dynamics of trypanosomes.

To the best of our knowledge, this study revealed for the first time *T. b. gambiense* in animals from HAT foci in Chad, particularly in horses and donkeys. These results suggest horses and donkeys as potential reservoirs of *T. b. gambiense* in HAT foci in Chad. Interestingly, no animals from the Mandoul and Moissala HAT foci were found with infection with *T. b. gambiense* and only three infections found in horses and donkeys were detected in the HAT focus of Maro. These results could be explained by vector control which was initiated in 2013 in some HAT foci through the deployment of tiny targets for the reduction of tsetse density and consequently, the trypanosomes’ transmission [50]. Compared to other HAT foci in Chad, the Maro focus reports the highest number of *T. b. gambiense* infections in humans. When the results of *T. b. gambiense* infections in humans and animals are put together, it appears that the human-infective trypanosome was found in animals when the disease prevalence was high in humans. These observations contradict those of other HAT foci, where *T. b. gambiense* was detected in animals of HAT foci showing low to very low disease prevalence [11, 15, 16, 51–53]. The discrepancies between these results could be linked to animal species as well as the epidemiological patterns in each focus. In our study, horses and donkeys are regularly in close contact with humans, and therefore could be more subjected to tsetse flies that have fed on humans infected with trypanosomes. In such context, these animals are more likely to be involved in the transmission cycle involving humans (human-tsetse-horses/donkey). In other studies, the animals identified as a potential reservoir for *T. b. gambiense* are more likely involved in the animal transmission cycle [15, 16, 51–53]. Investigations on tsetse blood meals from different HAT foci could improve the understanding of the contact frequency between tsetse and such animals. The epidemiology implications of these animals may vary according to the epidemiological patterns of each HAT focus.

## Conclusions

This study revealed high natural trypanosome infection rate and several trypanosome species and subspecies in donkeys and horses of HAT foci in Chad. The absence of a significant difference in the infection rate of different trypanosome species or subspecies suggests similar transmission patterns of trypanosomes in these HAT foci. The identification of *T. b. gambiense* in donkeys and horses suggests that these animals are potential reservoirs of human-infective trypanosomes in Chad. These animals must be taken into account for refining control strategies aiming to eliminate and interrupt HAT transmission. The identification of several animal trypanosomes as well as human-infective trypanosomes highlights the need for developing control strategies to fight HAT and AAT, with the overarching goal of improving animal and human health.

## Supplementary information

**Additional file 1: Table S1.** Concordance between CTC and RDT.

**Additional file 2: Table S2.** Concordance between RDT and PCR targeting all trypanosome species.

**Additional file 3: Table S3.** Concordance between RDT and PCR targeting *T. b. gambiense.*

## Data Availability

All data generated and/or analyzed during this study are included in the article and its additional files.

## Abbreviations

HAT: human African trypanosomiasis
AAT: animal African trypanosomiasis
WHO: World Health Organization
PCR: polymerase chain reaction
RDT: rapid diagnostic test
CTC: capillary tube centrifugation
CTAB: cethyl trimethyl ammonium bromide
ITS: internal transcribed spacer
UV: ultraviolet
