# Supplementary material for: Trypanosome infections in naturally infected horses and donkeys of three active sleeping sickness foci in the south of Chad
Source: Parasit Vectors. 2020 Jun 23;13:323. doi: 10.1186/s13071-020-04192-1 (PMC7310289; doi:10.1186/s13071-020-04192-1)
Supplement: Supplementary file 1 — Additional file 1: Table S1. Concordance between CTC and RDT. [file 13071_2020_4192_MOESM1_ESM.docx]

**Additional file 1: Table S1.** Concordance between CTC and RDT

|  | CTC^+^ | CTC^-^ | Total |
| --- | --- | --- | --- |
| RDT^+^ | 25 | 29 | 54 |
| RDT^-^ | 11 | 221 | 232 |
| Total | 36 | 250 | 286 |
