# Supplementary material for: Trypanosome infections in naturally infected horses and donkeys of three active sleeping sickness foci in the south of Chad
Source: Parasit Vectors. 2020 Jun 23;13:323. doi: 10.1186/s13071-020-04192-1 (PMC7310289; doi:10.1186/s13071-020-04192-1)
Supplement: Supplementary file 2 — Additional file 2: Table S2. Concordance between RDT and PCR targeting all trypanosome species. [file 13071_2020_4192_MOESM2_ESM.docx]

**Additional file 2: Table S2.** Concordance between RDT and PCR targeting all trypanosome species

|  | PCR^+^ | PCR^-^ | Total |
| --- | --- | --- | --- |
| RDT^+^ | 38 | 16 | 54 |
| RDT^-^ | 63 | 169 | 232 |
| Total | 101 | 185 | 286 |
