# Supplementary material for: Trypanosome infections in naturally infected horses and donkeys of three active sleeping sickness foci in the south of Chad
Source: Parasit Vectors. 2020 Jun 23;13:323. doi: 10.1186/s13071-020-04192-1 (PMC7310289; doi:10.1186/s13071-020-04192-1)
Supplement: Supplementary file 3 — Additional file 3: Table S3. Concordance between RDT and PCR targeting T. b. gambiense. [file 13071_2020_4192_MOESM3_ESM.docx]

**Additional file 3: Table S3.** Concordance between RDT and PCR targeting *T. b. gambiense*

|  | TBG^+^ | TBG^-^ | Total |
| --- | --- | --- | --- |
| RDT^+^ | 3 | 51 | 54 |
| RDT^-^ | 0 | 232 | 232 |
| Total | 3 | 283 | 286 |
